# Zone 2.5 TEVAR with L-Shaped Marker-Guided Fenestration for Stanford Type B Aortic Dissection

**DOI:** 10.3400/avd.cr.25-00090

**Published:** 2025-10-25

**Authors:** Norimasa Haijima, Mikihiko Kudo, Satoru Murata, Takuya Ono, Hideyuki Shimizu

**Affiliations:** 1 Department of Cardiovascular Surgery, National Hospital Organization Saitama Hospital, Wako, Saitama, Japan; 2 Department of Cardiovascular Surgery, Keio University Hospital, Tokyo, Japan

**Keywords:** TEVAR, PMEG, aortic dissection

## Abstract

A patient with complicated Stanford type B aortic dissection and a large ulcer-like projection just distal to the left subclavian artery (LSA) underwent thoracic endovascular aortic repair (TEVAR) using a physician-made 1-cm fenestration and L-shaped marker. This technique allowed accurate alignment with the LSA under fluoroscopic guidance without additional devices. Postoperative and 6-month follow-up computed tomography confirmed good outcomes. This simplified, economical Zone 2.5 TEVAR approach may be a viable treatment option for high-risk patients with anatomically challenging aortic dissections.

## Introduction

Thoracic endovascular aortic repair (TEVAR) is a well-established, minimally invasive treatment strategy for Stanford type B aortic dissection (TBAD). In cases of complicated TBAD, such as those with rapid expansion of the false lumen or formation of ulcer-like projections (ULPs), early intervention with TEVAR is recommended.^1)^

However, when the lesion extends just distal to the left subclavian artery (LSA), securing an adequate landing zone becomes technically challenging. In such cases, LSA preservation or reconstruction is often required during Zone 2 deployment. Techniques such as surgical bypass, chimney technique, *in situ* fenestration (ISF), or physician-modified endografts (PMEGs) have been reported; however, these approaches are associated with increased procedural complexity, cost, and potential device-related complications.^2–4)^

Herein, we report a case of complicated TBAD with a large ULP just distal to the LSA, successfully treated with Zone 2.5 TEVAR using a physician-made fenestration and an additional L-shaped marker to enhance graft orientation under fluoroscopic guidance.

In this report, we use the term “Zone 2.5” to describe deployment at the mid-portion of the LSA origin with a physician-modified fenestration. Although this term is not part of the standard classification, it is used here for clarity.

## Case Report

An 86-year-old male residing in a nursing care facility presented with sudden-onset chest and back pain. His medical history included old cerebral infarction, hypertension, hyperuricemia, and left carotid artery stenosis. He was receiving cilostazol (Pletaal) 100 mg twice daily for antiplatelet therapy.

**Fig. 1:**
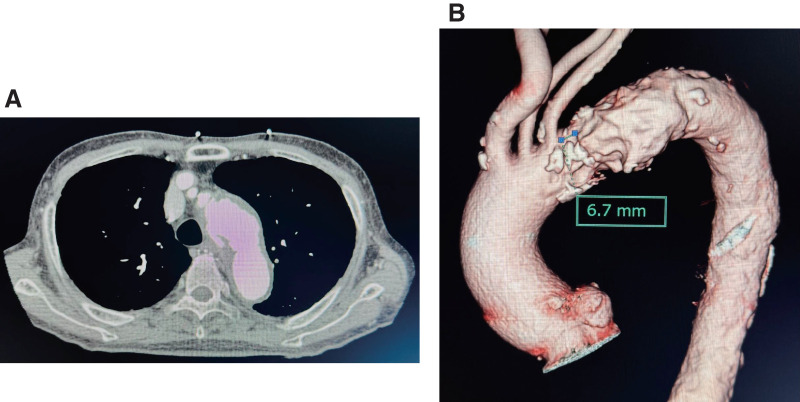
(**A**) Preoperative contrast-enhanced CT (axial view) showing a large ulcer-like projection just distal to the left subclavian artery. (**B**) Preoperative 3D CT indicating a proximal landing zone of 6.7 mm. 3D: 3-dimensional; CT: computed tomography

Contrast-enhanced computed tomography (CT) revealed a thrombosed Stanford TBAD with a large ULP just distal to the LSA, along with a 50-mm abdominal aortic aneurysm (AAA). The patient was admitted to the intensive care unit for conservative management with antihypertensive therapy. However, follow-up CT on day 7 demonstrated rapid expansion of the ULP, and the case was diagnosed as complicated TBAD (**Fig. 1**).

Given the patient’s advanced age, progressive ULP, and concomitant AAA, we elected to perform simultaneous TEVAR and endovascular abdominal aortic repair (EVAR). The entry tear was located immediately distal to the LSA, resulting in a short proximal landing zone of 6.7 mm, which necessitated preservation of LSA flow and precise graft deployment. Although debranching TEVAR and Viabahn (W. L. Gore & Associates, Flagstaff, AZ, USA)-assisted fenestration TEVAR were considered, we selected a physician-modified fenestrated TEVAR with a Zone 2.5 deployment, prioritizing technical simplicity and cost-effectiveness. We avoided debranching because of the patient’s frailty and increased surgical risk, and Viabahn-assisted fenestration was not selected owing to the additional cost and complexity. In contrast, the physician-modified approach with an L-shaped marker enabled rapid and precise alignment while minimizing invasiveness, making it the most appropriate option for this high-risk case.

The stent graft used was the Valiant Thoracic (VAMF3232C150TJ; Medtronic, Santa Rosa, CA, USA). Preoperative CT revealed an aortic diameter of 29 mm just distal to the LSA, and a 32-mm graft was chosen to allow for approximately 10% oversizing.

A 1-cm square fenestration was created in the non-stented portion of the graft, targeting the bare stent segment and avoiding the fixation sutures. Using a disposable surgical scalpel, we incised the graft fabric without structural damage.

**Fig. 2:**
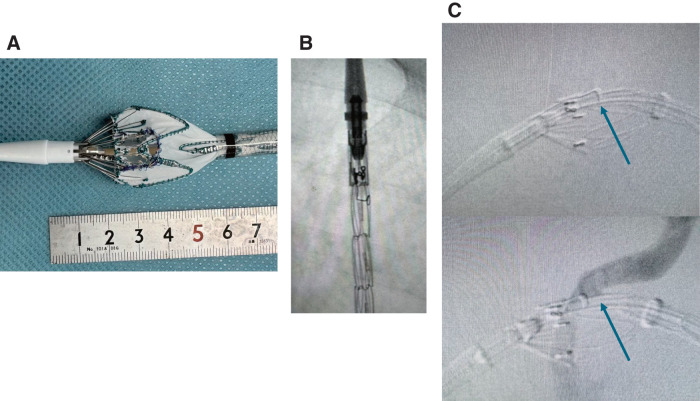
(**A**) Physician-modified stent graft with a 1-cm square fenestration reinforced by an L-shaped radiopaque marker. (**B**) Intraoperative fluoroscopy demonstrating clear visibility of the L-shaped marker during advancement in the descending aorta. (**C**) Angiography from the LSA during deployment, showing accurate alignment of the fenestration with the mid-portion of the LSA origin (Zone 2.5). LSA: left subclavian artery

To enhance fluoroscopic visibility, we prepared an L-shaped radiopaque marker from the distal tip of a Micropuncture introducer wire (Cook Medical, Bloomington, IN, USA) and secured it along the fenestration margin with a running 5-0 Prolene suture (Ethicon, Somerville, NJ, USA) (**Fig. 2A**).

During intraoperative fluoroscopy, the L-shaped marker was clearly visible when the device was advanced into the descending aorta, providing straightforward confirmation of the graft’s rotational orientation (**Fig. 2B**). At the time of deployment, angiography from the LSA demonstrated accurate alignment of the fenestration with the mid-portion of the LSA origin (Zone 2.5), with the L-shaped marker serving as a reliable landmark (**Fig. 2C**).

A 5-Fr sheath was inserted *via* the left radial artery, and a pigtail catheter was advanced through the LSA into the ascending aorta. Under fluoroscopic guidance, the positional alignment of the fenestration with the LSA origin was confirmed. The stent graft was deployed in Zone 2, positioning the lower edge of the L-shaped marker at the mid-portion of the LSA origin, corresponding to our definition of a Zone 2.5 deployment. Post-deployment angiography confirmed preserved perfusion to the LSA through the fenestration, without the need for additional devices (e.g., a Viabahn stent). EVAR was then successfully performed. Total operative time was 2 hours and 33 minutes, with 49 minutes of fluoroscopy and 107 mL of contrast used.

**Fig. 3:**
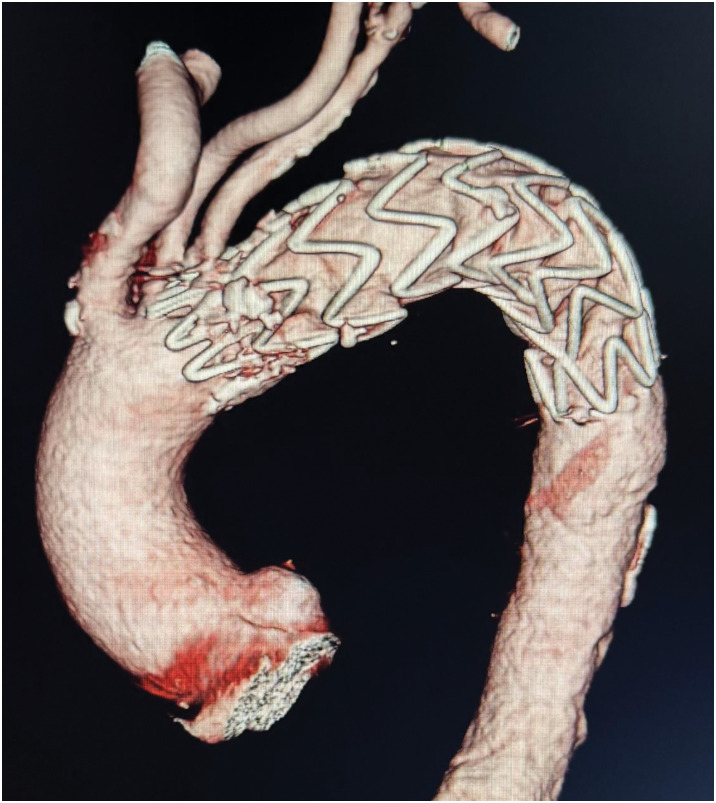
Postoperative contrast-enhanced CT demonstrating complete coverage of the ULP and preserved perfusion of the LSA. CT: computed tomography; LSA: left subclavian artery; ULP: ulcer-like projection

The postoperative course was uneventful, with no neurological deficits, limb dysfunction, encephalopathy, or signs of left upper limb ischemia. Early postoperative CT confirmed exclusion of the ULP and preserved LSA perfusion (**Fig. 3**). At 6-month follow-up, CT showed maintained LSA patency without endoleak or need for reintervention.

## Discussion

In Stanford TBAD, the presence of a patent false lumen or progression of an ULP increases the risk of rupture, making early TEVAR the recommended treatment.^1)^ When the lesion is located just distal to the LSA, Zone 2 deployment often necessitates preservation or reconstruction of the LSA. While conventional techniques like debranching TEVAR, the chimney technique, ISF, and Viabahn-assisted fenestrated TEVAR are available,^2–6)^ they are associated with increased procedural complexity, additional device costs, and a higher potential for complications.

ISF can avoid rotational alignment issues by creating the fenestration *in situ*, whereas PMEGs require precise orientation.^7)^ To address these limitations, we adopted a simplified Zone 2.5 strategy that combines a physician-made fenestration with an L-shaped radiopaque marker.

In this case, we used a Zone 2.5 deployment strategy with a physician-made fenestration. We created a 1-cm square fenestration in the graft’s non-stented portion using a disposable scalpel, carefully avoiding the bare stent’s fixation sutures. To improve fluoroscopic visualization, we sutured an L-shaped radiopaque marker, made from the distal tip of a Micropuncture introducer wire, along the fenestration’s margin with a running 5-0 Prolene suture. During deployment, the marker’s lower edge was positioned at the mid-portion of the LSA origin, providing a clear landmark for alignment. This technique allowed for accurate fenestration placement without the need for rapid pacing or additional sophisticated devices.

Recent studies have highlighted the importance of visibility and rotational alignment of fenestrations in both ISF and PMEGs.^2–5,7)^ Our approach, which uses a body-compatible and low-cost radiopaque wire, provides a simple and reproducible solution for enhancing graft orientation without requiring a custom-made marker. The key to precise deployment lies in detailed preoperative imaging, particularly 3-dimensional CT analysis to measure the course and length of the LSA, as well as intraoperative repetition of contrast injections from the radial approach to guide final alignment.

The L-shaped marker offers significant technical advantages compared with the straight or circular radiopaque markers commonly used in PMEG. Its geometry provides 2 perpendicular reference axes and a single, easily discernible vertex that remains conspicuous even when partially superimposed on graft struts. This enables immediate recognition of the fenestration’s rotational orientation while the device is still in the descending thoracic aorta, simplifying “clock-face” alignment before negotiating the arch. Under fluoroscopy, we use complementary projections: the left anterior oblique view to judge anteroposterior depth relative to the arch and the right anterior oblique view to confirm right–left alignment with the LSA origin. The visible corner of the L therefore serves as an unambiguous landmark in both planes. As a practical tip, preloading the delivery system with the fenestration oriented at approximately the 5-o’clock position at insertion biases the fenestration cranially toward the LSA during advancement. In the present case, this simple geometry improved confidence in intraoperative positioning and obviated the need for additional alignment devices.

This method also allowed LSA preservation without adjunctive devices like Viabahn, significantly reducing procedural costs. In Japan, Viabahn stents are not reimbursed and must be funded by the hospital, whereas a Micropuncture wire costs only a fraction of that amount. In addition to cost savings, avoiding Viabahn eliminates the risks of stent occlusion and type III endoleak reported in previous series.^4,5)^ Furthermore, because the fenestration remains accessible and aligned with the bare stent, secondary interventions are technically easier compared to covered chimney or ISF procedures.^6)^

The size of the 1-cm square fenestration was determined based on preoperative CT findings. Its vertical dimension was aligned with the length of the LSA orifice, and its horizontal width was set to approximately 60% of the diameter. This proportion was selected to orient the fenestration slightly anteriorly, thereby reducing the risk of inadvertent coverage of the left common carotid artery. This design ensured sufficient perfusion while preserving adequate graft membrane for balloon apposition, thereby achieving hemostasis and structural integrity.

The concept of Zone 2.5 represents an anatomical midpoint between Zones 2 and 3, allowing for LSA preservation while maximizing the proximal sealing length.

This approach is particularly applicable in cases where the distance from the LSA to the lesion is ≥7–10 mm, which provides an adequate sealing zone of at least 15 mm when combined with the fenestrated segment.

For borderline cases with a distance of around 6 mm, careful preoperative imaging and intraoperative angiographic confirmation are required, as technical feasibility may vary depending on patient anatomy.

If the lesion is located within approximately 5 mm of the LSA origin, the available proximal landing zone may be insufficient to ensure safe and stable graft deployment. In such cases, the Zone 2.5 strategy may not be feasible, and alternative approaches such as debranching or ISF should be considered.

Previous studies by Chastant et al., Queiroz et al., and Matsumoto et al. have also highlighted the importance of fenestration accuracy and visibility in PMEG-based TEVAR.^4,5,7)^ Our technique, which combines an L-shaped radiopaque marker with practical intraoperative strategies, offers a reproducible and cost-effective alternative. This is especially valuable for elderly or high-risk patients for whom the use of additional devices is undesirable. However, this Zone 2.5 strategy is not suitable for all cases. Contraindications include lesions located within 5 mm of the LSA origin, severe vessel tortuosity, significant calcification, or stenosis of the LSA origin. While the 6-month follow-up demonstrated favorable outcomes, long-term risks such as type III endoleak from the fenestration site or marker displacement must be considered. In the present case, no complications were observed at 6 months, although the radiopaque marker was barely visible on contrast-enhanced CT.

Ongoing evaluation is warranted to assess long-term durability and safety. To operationalize this, we instituted a predefined surveillance protocol: contrast-enhanced CT at 12 and 24 months and annually thereafter, complemented by bilateral brachial blood pressure measurements and targeted duplex ultrasonography of the LSA origin to screen for stenosis or subclavian steal. When iodinated contrast is undesirable, non-contrast CT combined with duplex will be substituted, with selective contrast studies as indicated. Reintervention will be considered upon evidence of type III endoleak at the fenestration site, progressive LSA stenosis/occlusion, stent graft migration, or unfavorable aortic remodeling.

## Conclusion

This case demonstrates that Zone 2.5 TEVAR using a physician-made fenestration with an L-shaped radiopaque marker allowed safe, accurate deployment without additional devices in a patient with complicated type B dissection with a large ULP immediately distal to the LSA. This simplified approach may serve as a reproducible, cost-effective option in anatomically challenging cases. We have implemented annual imaging surveillance (contrast-enhanced CT or duplex-based alternatives) to monitor long-term durability and guide timely reintervention if needed.

## References

[1] Isselbacher EM, Preventza O, Hamilton Black J 3rd, et al. 2022 ACC/AHA guideline for the diagnosis and management of aortic disease: a report of the American Heart Association/American College of Cardiology Joint Committee on clinical practice guidelines. Circulation 2022; 146: e334–482. 36322642 10.1161/CIR.0000000000001106 PMC9876736

[2] Redlinger RE Jr, Ahanchi SS, Panneton JM. In situ laser fenestration during emergent thoracic endovascular aortic repair is an effective method for left subclavian artery revascularization. J Vasc Surg 2013; 58: 1171–7. 23746832 10.1016/j.jvs.2013.04.045

[3] Li G, Li M, Dong Z, et al. In situ needle fenestration for aortic arch conditions during thoracic endovascular aortic repair. BMC Cardiovasc Disord 2024; 24: 625. 39511486 10.1186/s12872-024-04322-y PMC11545571

[4] Chastant R, Belarbi A, Ozdemir BA, et al. Homemade fenestrated physician-modified stent grafts for arch aortic degenerative aneurysms. J Vasc Surg 2022; 76: 1133–40.e2. 35697312 10.1016/j.jvs.2022.04.041

[5] Queiroz AB, Lopes JB, Santos VP, et al. Physician-modified endovascular grafts for zone-2 thoracic endovascular aortic repair. Aorta (Stamford) 2022; 10: 13–9. 35640582 10.1055/s-0042-1742696 PMC9179216

[6] Xie W, Xue Y, Li S, et al. Left subclavian artery revascularization in thoracic endovascular aortic repair: single center’s clinical experiences from 171 patients. J Cardiothorac Surg 2021; 16: 207. 34330305 10.1186/s13019-021-01593-w PMC8325210

[7] Canaud L, Morishita K, Gandet T, et al. Homemade fenestrated stent-graft for thoracic endovascular aortic repair of zone 2 aortic lesions. J Thorac Cardiovasc Surg 2018; 155: 488–93. 28867380 10.1016/j.jtcvs.2017.07.045

